# Estimation of Vibration Characteristics of a Space Manipulator From Air Bearing Supported Test Data

**DOI:** 10.3389/frobt.2021.641165

**Published:** 2021-05-13

**Authors:** Haiquan Li, Qingqing Wei, Jianxun Liang, Weiyan Ren, Zixin Tang, Delun Li

**Affiliations:** ^1^School of Aerospace Engineering, Tsinghua University, Beijing, China; ^2^Beijing Key Laboratory of Intelligent Space Robotic System Technology and Applications, Beijing, China; ^3^Beijing Institute of Spacecraft System Engineering, Beijing, China

**Keywords:** space manipulator, air bearing, vibration, ground-test, identification

## Abstract

Space manipulators have attracted much attention due to their implications in on-orbit servicing in recent years. Air bearing based support equipment is widely used for ground test to offset the effect of gravity. However, an air bearing support introduces a new problem caused by additional inertial and mass properties. Additional mass and inertial load will influence the dynamics behavior, especially stiffness information and vibration response of the whole ground test system. In this paper, a set of procedures are presented to remove the influence of air bearings and identify the true equivalent joint stiffness and damping from the test data of a motor-braked space manipulator with an air bearing support. First, inertia parameters are identified. Then, the equivalent joint stiffness and damping are determined by using a genetic algorithm (GA) method. Finally, true vibration characteristics of the manipulator are estimated by removing the additional inertia caused by the air bearings. Moreover, simulations and experiments are carried out to validate the presented procedures.

## 1. Introduction

Space manipulator systems, of the kinds used in space missions, have attracted much attention due to their high performance in active debris removal (Shan et al., 2016) and on-orbit servicing (Flores-Abad et al., 2014) in recent years. Over the past decades, a succession of technological advances has been made in both hardware device designs (Yoshida, 2009; Jaekel et al., 2018) and software algorithm developments (Nanos and Papadopoulos, 2017; Valverde and Tsiotras, 2018; Virgili-Llop and Romano, 2019; Liu et al., 2020).

Space manipulator systems have high requirements for safety and reliability on account of the fact that operational errors of the manipulator may cause serious damage to the system. Consequently, in order to reduce the risk of on orbit operations, strict ground tests before launch are indispensable for both hardware and software. Among the existing ground test facilities, the air bearing testbed has been widely used due to its simple structure, long simulation time, and the least influence of resistance and reaction force (Wilde et al., 2019). Many air bearing testbeds have been built and used for hardware testing (Rybus and Seweryn, 2016; Mantellato et al., 2017) and control algorithm verification (Cocuzza et al., 2010, 2011; Rybus et al., 2013) in zero-gravity condition. Functional prototypes were designed and tested by air bearing facilities for various applications. Most of new end-effectors used for self-relocation (Han et al., 2016), target capturing (Liu et al., 2015; Kwok Choon et al., 2018), surface sampling (Moreland et al., 2018), and spacecraft refueling (Medina et al., 2017) were tested and verified carefully before on-orbit applications. Moreover, many algorithmic procedures have been presented and verified on an air bearing testbed for theoretical research such as capturing controller design Huang et al. (2018), on-orbit parameters identification and calibration (Li et al., 2017; Meng et al., 2020), and trajectory planning for space manipulators (Sabatini et al., 2017).

The studies mentioned above have provided considerable results about air bearing experiments. However, most of the existed experiments ignored the additional mass and inertia of the supporting air bearings, which will significantly affect the dynamic characteristics of the manipulator system during air bearing test tasks, i.e., joints torque, and vibrations measured in an air bearing test are different from those in a similar on-orbit task. The influence of the air bearings on joint torque was studied and decoupled from air bearing test data (Ma and Zhao, 2015; Yao et al., 2018). On the other hand, there is no open literature about the influence on air bearing tested vibration characteristics. It is generally accepted that the flexibility of a manipulator cannot be avoided completely. In practice, flexibility will improve the adaptability of the end effector, which ensures the manipulator is not easily damaged. However, flexibility will also include unexpected oscillations, which will significantly increase the propellant expenditure and influence the control precision (Virgili-Llop et al., 2017). In actual on-orbit tasks, especially when a space manipulator has finished performing trajectories and brakes, residual vibrations will be observed (Meng et al., 2018; Ren et al., 2018). Moreover, it takes astronauts about 20 to 40s to wait for the attenuation of vibration excited during the process of manipulator movement before the next operation (Meng et al., 2018). So, an estimation of the true vibration characteristics from ground test data is very important for on-orbit task design.

In this paper, vibration characteristics of an air bearing supported manipulator with a flexible joint are studied. The aim of the presented work is to develop a decouple procedure by which the real on-orbit vibration characteristics can be determined from the air bearing test data of a motor-braked manipulator. Dynamic equations of the air bearing test system are established, and a three-step procedure is developed to remove the dynamics effect of supporting air bearings. First, inertia parameters of the manipulator with air bearings are identified. Then the equivalent joint stiffness and damping are determined by using a genetic algorithm (GA) method. Finally, true vibration characteristics of the manipulator are estimated by removing the additional inertia caused by the air bearings. The proposed method is verified by a set of numerical simulations and air bearing experiments.

The rest of the presented work is organized as follows. In section 2, dynamic equations of the system is established, and a three-step procedure including parameter identification and additional inertia removing is presented in detail. Concequently, simulations and experiments are conducted to verify the presented method in section 3. Finally, the paper is summarized in section 4.

## 2. Materials and Methods

Dynamics model of a motor-braked manipulator supported by air bearings is established in this section, based on which a three-step procedure is developed to remove the influence of air bearings. In this study, a dimension reduced equivalent manipulator with joint flexibility is adopted to illustrate the established method.

### 2.1. Dynamics Equation

**Figure 1:**
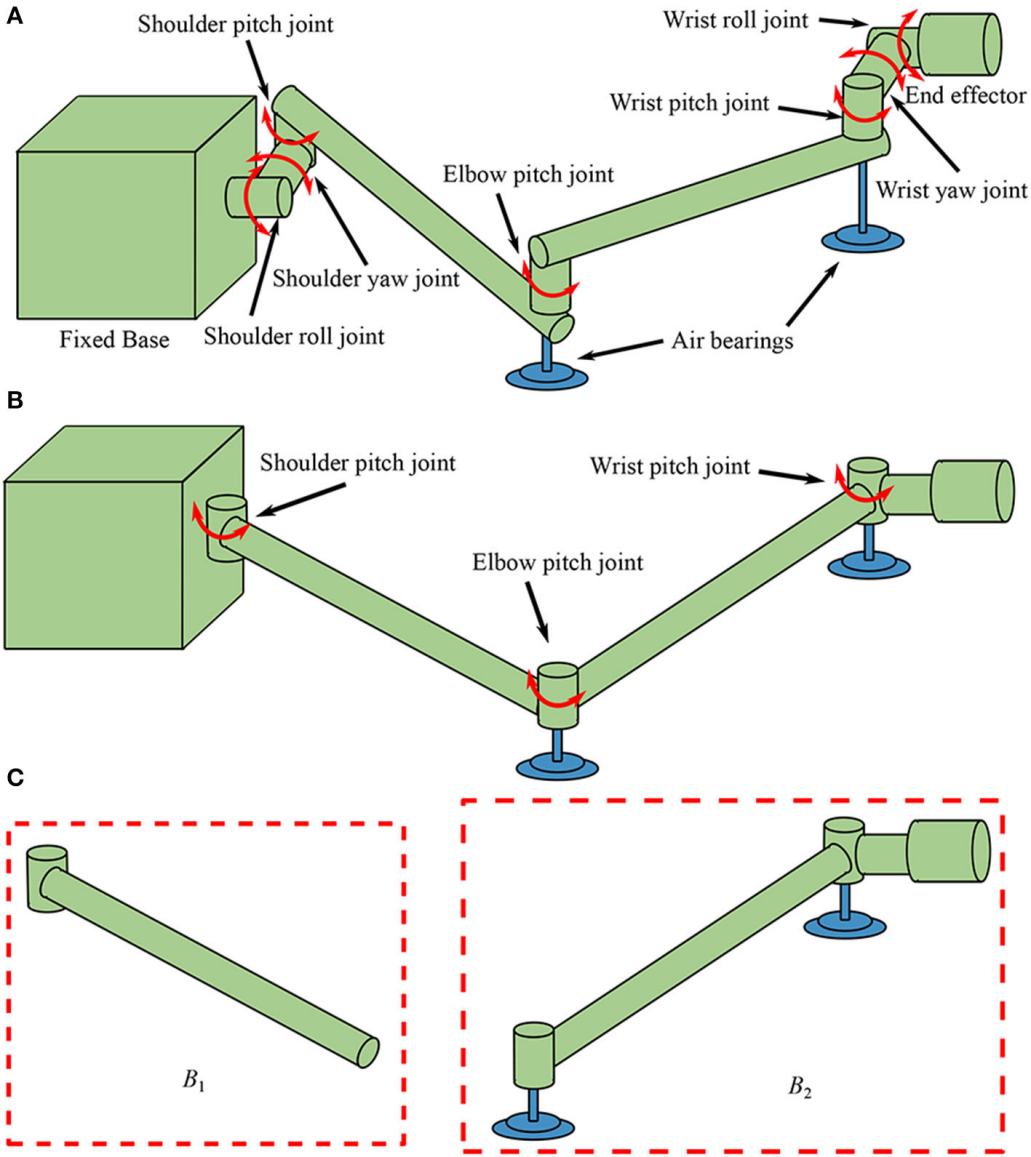
Manipulators used for air bearing test: **(A)** a 7-DOF manipulator, **(B)** a reduced equivalent manipulator, and **(C)** the two composite bodies.

In order to test complex tasks in on-orbit operations, a “section” method is usually used as a practical option (Liu et al., 2015). With this method, first, a series of planar motions are tested, and then the composition of the planar motions is treated as a spatial one. Therefore, dimension reduced dynamics equivalent models in a plane are often used in the air bearing test facilities (Du et al., 2018). A typical 7-DoF manipulator is illustrated in Figure 1A for air bearing ground test. Due to the planar motion constraint, the four yaw and roll joints are constrained, and a reduced equivalent manipulator shown in Figure 1B is usually used to replace the 7-DOF one in the air bearing facilities.

A flexible-link manipulator and a rigid-link manipulator are designed for different tasks. Manipulators with flexible links have good robustness but poor positioning accuracy and are suitable for heavy load transportation or auxiliary docking. Rigid-link manipulators have high precision and are suitable for fine operation and target capture. This paper mainly studies the joint vibration of rigid-link manipulators such as ETS-VII and the Orbital Express. Equivalent stiffness and damping are lumped at the joint level in this study, and links are simplified as rigid ones (Nanos and Papadopoulos, 2015).

**Figure 2:**
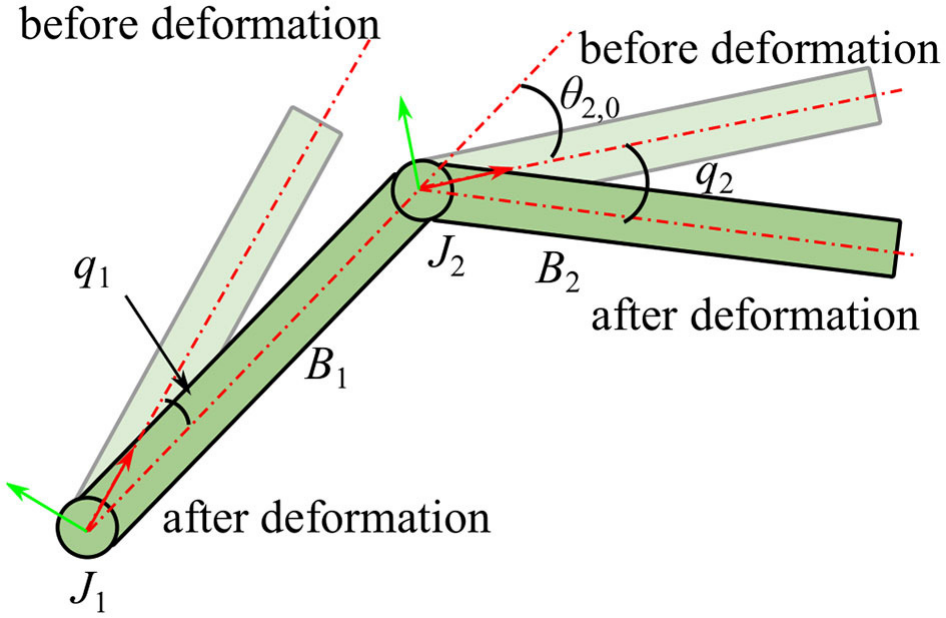
Coordinate system of the manipulator.

It is assumed that the manipulator is fixed on a large base such as the International Space Station (ISS) so that the reaction motion of the base caused by the manipulator can be ignored, thus, the equivalent manipulator shown in Figure 1B consists of three joints and two links. From the base to the end-effector, the three joints are named as shoulder joint (*J*_1_), elbow joint (*J*_2_), and wrist joint (*J*_3_), separately. The upper arm link *L*_1_ is connected to the base by *J*_1_, the lower arm link *L*_2_ is connected to *L*_1_ by *J*_2_, and the end-effector is connected to *L*_2_ by *J*_3_. In this equivalent manipulator system, displacements of the end-effector are mainly determined by the first two joints while the wrist joint is mainly used to adjust the orientation of the end-effector. Therefore, vibrations of the motor-braked manipulator are mainly caused by flexibilities of the first two joints, so the flexibility of *J*_3_ is neglected in this work. Consequently, the system is separated into two composite bodies *B*_1_ and *B*_2_ as shown in Figure 1C. The equivalent angular deformations *q*_1_ and *q*_2_ are chosen to be the generalized coordinates, and reference coordinate system is established as shown in Figure 2. The dynamics equation of the system is obtained as:

(1) $$\underline{Z}\underline{\ddot{q}}+{\underline{f}}^{w}+{\underline{f}}^{k}=\underline{0}$$

where *Z* is the generalized mass matrix, *f*^*w*^ is the non-linear torque caused by inertia, and *f*^*k*^ is a set of joint passive torque including stiffness and damping. Due to the fact that the joint motors are braked, the motor driven torque vanishes in Equation (1). The generalized mass matrix *Z* can be expanded as:

(2) $$\begin{array}{l} \;\underline{Z}\\ =\left(\begin{array}{cc} I_{1}+I_{2}+m_{2}l_{1}^{2}+2l_{1}m_{2}[\rho_{2}\cos(\theta_{2,0}+q_{2})] & m_{2}\rho_{2}l_{1}\cos(\theta_{2,0}+q_{2})+I_{2}\\ m_{2}\rho_{2}l_{1}\cos(\theta_{2,0}+q_{2})+I_{2} & I_{2} \end{array}\right) \end{array}$$

where *I*_1_ is the inertia of *B*_1_ expressed in the body fixed frame and *I*_2_ is the inertia of *B*_2_ in the body fixed frame. *l*_1_ and *l*_2_ depict the length of the two links *L*_1_ and *L*_2_. *m*_2_ is the mass of *B*_2_. *ρ*_2_ is the axial coordinate of the mass center of *B*_2_ decomposed in the body fixed frame. The value of *ρ*_2_ is usually unknown due to the asymmetric of *B*_2_. *θ*_2, 0_ is the initial angle of *J*_2_.

The non-linear inertia torque *f*^*w*^ is given by:

(3) $$\begin{array}{l} {\underline{f}}^{w}=\left(\begin{array}{c} m_{2}l_{1}\left({\dot{q}}_{2}^{2}-{\dot{q}}_{1}^{2}\right)\sin\left(\theta_{2,0}+q_{2}\right)\rho_{2}\\ -m_{2}l_{1}\dot{q_{1}^{2}}\sin\left(\theta_{2,0}+q_{2}\right)\rho_{2} \end{array}\right) \end{array}$$

where ${\dot{q}}_{1}$ and ${\dot{q}}_{2}$ depict the angular velocities of *J*_1_ and *J*_2_, respectively. For constant coefficients of stiffness and damping, the joint passive torque *f*^*k*^ is given by:

(4) $$\begin{array}{l} {\underline{f}}^{k}=\left(\begin{array}{c} k_{1}q_{1}+c_{1}{\dot{q}}_{1}\\ k_{2}q_{2}+c_{2}{\dot{q}}_{2} \end{array}\right) \end{array}$$

where *k*_*i*_ and *c*_*i*_, (*i* = 1, 2) are the stiffness and damping coefficients of *J*_*i*_, (*i* = 1, 2).

In order to estimate the true vibration characteristics of the manipulator, the primary effect introduced by air bearings should be determined. Inertial parameters affected by the air bearings can be expressed as:

(5) $$\{\begin{array}{c} m_{2}=m_{2,0}+m_{s_{1}}+m_{s_{2}}\\ I_{2}=I_{2,0}+I_{s_{1}}+I_{s_{2}}+m_{s_{2}}l_{2}^{2}\\ m_{2}\rho_{2}=m_{2,0}\rho_{2,0}+m_{s_{2}}l_{2} \end{array}$$

where *m*_*si*_, (*i* = 1, 2) and *I*_*si*_ are the mass and inertia of the *i*-th air bearing.

As can be seen in Equations (1, 5), the extra terms in the dynamics equation are caused by additional inertia of the air bearings. Therefore, a primary route to get the true dynamics information is to identify the dynamics parameters of the air bearing supported system, and then remove the additional inertia of the air bearings. So, an essential step is to identify the parameters of the air bearing supported manipulator system.

### 2.2. Identifiability of the Parameters

For parameter identification of a system presented by Equation (1), if values of the mass, inertia, stiffness and damping are scaled by an arbitrary constant factor *α*, the three terms in Equation (1) can be rewritten as:

(6) $$\left(\begin{array}{cc} \alpha I_{1}+\alpha I_{2}+\alpha m_{2}l_{1}^{2}+2l_{1}\alpha m_{2}[\rho_{2}\cos(\theta_{2,0}+q_{2})] & \alpha m_{2}\rho_{2}l_{1}\cos(\theta_{2,0}+q_{2})+\alpha I_{2}\\ \alpha m_{2}\rho_{2}l_{1}\cos(\theta_{2,0}+q_{2})+\alpha I_{2} & \alpha I_{2} \end{array}\right)=\alpha \underline{Z}$$

(7) $$\begin{array}{l} \left(\begin{array}{c} \alpha m_{2}l_{1}\left({\dot{q}}_{2}^{2}-{\dot{q}}_{1}^{2}\right)\sin\left(\theta_{2,0}+q_{2}\right)\rho_{2}\\ -\alpha m_{2}l_{1}\dot{q_{1}^{2}}\sin\left(\theta_{2,0}+q_{2}\right)\rho_{2} \end{array}\right)=\alpha {\underline{f}}^{w} \end{array}$$

(8) $$\begin{array}{l} \left(\begin{array}{c} \alpha k_{1}q_{1}+\alpha c_{1}{\dot{q}}_{1}\\ \alpha k_{2}q_{2}+\alpha c_{2}{\dot{q}}_{2} \end{array}\right)=\alpha {\underline{f}}^{k} \end{array}$$

Therefore, dynamics equation of the scaled system is:

(9) $$\alpha (\underline{Z}\ddot{\underline{q}}+{\underline{f}}^{w}+{\underline{f}}^{k})=\underline{Z}\ddot{\underline{q}}+{\underline{f}}^{w}+{\underline{f}}^{k}=\underline{0}$$

It is obvious that different sets of parameters lead to the same dynamics equation, which indicates that only the proportional relationship between the joint parameters and inertia can be determined by motion data of a system described by Equation (1). In order to complete the identification, inertial parameters should be determined at first. Then, corresponding joint information could be identified. As a result, a three-step procedure is presented as follows:

1. Identify the inertial parameters by driving the joints according to a prescribed trajectory.
2. Identify the joint stiffness and damping with motion data and the inertial parameters identified from step one.
3. Remove the additional inertia of the air bearings and establish the dynamics equation with the new inertial parameters and the joint parameters determined by step 2.

Details of these steps will be presented in section 2.3.

### 2.3. Identification of Inertia

The inertia of a composite part can be identified by the least-squares method, which is usually used to identify the inertia information of an industrial manipulator (Wu et al., 2010). When the shoulder joint *J*_1_ is locked, the relationship between motor torque and motions of the other joint *J*_2_ can be written as:

(10) $$\begin{array}{l} I_{2}{\ddot{q}}_{2}-f_{f_{2}}\text{sign}\left({\dot{q}}_{2}\right)-f_{v_{2}}{\dot{q}}_{2}=\tau_{2} \end{array}$$

and when *J*_2_ is locked, dynamics equation of the combination of *B*_1_ and *B*_2_ can be presented as:

(11) $$\begin{array}{l} \left(I_{1}+I_{2}+m_{2}l_{1}^{2}+2m_{2}l_{1}\rho_{2}\cos\left(\theta_{2,0}\right)\right){\ddot{q}}_{1}-f_{f_{1}}\text{sign}\left({\dot{q}}_{1}\right)-f_{v_{1}}{\dot{q}}_{1}=\tau_{1} \end{array}$$

where *f*_*f*_*i*__, (*i* = 1, 2) is a constant part of the friction torque and *f*_*v*_*i*__ is a viscous friction coefficient. *τ*_*i*_ is the drive torque generated by the motor of *J*_*i*_.

With a time series of data, Equations (10, 11) can be rewritten as:

(12) $$\begin{array}{l} \left(\begin{array}{ccc} {\ddot{q}}_{1,t_{1}} & \text{sign}({\dot{q}}_{1,t_{1}}) & {\dot{q}}_{1,t_{1}}\\ \vdots & \vdots & \vdots \\ {\ddot{q}}_{1,t_{n}} & \text{sign}({\dot{q}}_{1,t_{n}}) & {\dot{q}}_{1,t_{n}} \end{array}\right)\left(\begin{array}{c} I_{1}+I_{2}+m_{2}l_{1}^{2}+2m_{2}l_{1}\rho_{2}\cos(\theta_{2,0})\\ f_{f_{1}}\\ f_{v_{1}} \end{array}\right)\\ \;=\left(\begin{array}{c} \tau_{1,t_{1}}\\ \vdots \\ \tau_{1,t_{n}} \end{array}\right) \end{array}$$

and

(13) $$\begin{array}{l} \left(\begin{array}{ccc} {\ddot{q}}_{2,t_{1}} & \text{sign}\left({\dot{q}}_{2,t_{1}}\right) & {\dot{q}}_{2,t_{1}}\\ \vdots & \vdots & \vdots \\ {\ddot{q}}_{2,t_{n}} & \text{sign}\left({\dot{q}}_{2,t_{n}}\right) & {\dot{q}}_{2,t_{n}} \end{array}\right)\left(\begin{array}{c} I_{2}\\ f_{f_{2}}\\ f_{v_{2}} \end{array}\right)=\left(\begin{array}{c} \tau_{2,t_{1}}\\ \vdots \\ \tau_{2,t_{n}} \end{array}\right) \end{array}$$

Using the least squares method, the values of the parameters can be determined.

In order to get all the four elements in the generalized mass matrix *Z*, another value $\theta_{0,2}^{\prime }$ of the initial angle of *J*_2_ can be chosen to repeat the identification of Equation (11). Then, the relationship between the inertial parameters and the identified results is obtained as follows:

(14) $$\{\begin{array}{c} I_{1}+I_{2}+m_{2}l_{1}^{2}+2m_{2}l_{1}\rho_{2}\cos(\theta_{2,0})=p_{1}\\ I_{1}+I_{2}+m_{2}l_{1}^{2}+2m_{2}l_{1}\rho_{2}\cos(\theta_{2,0}^{\prime })=p_{1}^{\prime }\\ I_{2}=p_{2} \end{array}$$

where *p*_1_, $p_{1}^{\prime }$, and *p*_2_ are composite inertia values obtained from the least-squares method. Thus, the term *m*_2_*ρ*_2_ in Equation (1) can be determined by:

(15) $$\begin{array}{l} 2\left[\cos\left(\theta_{2,0}\right)-\cos\left(\theta_{2,0}^{\prime }\right)\right]m_{2}l_{1}\rho_{2}=p_{1}-p_{1}^{\prime } \end{array}$$

This equation can be solved by choosing suitable values of *θ*_2, 0_ and $\theta_{2,0}^{\prime }$ to ensure that:

(16) $$\begin{array}{l} \left[\cos\left(\theta_{2,0}\right)-\cos\left(\theta_{2,0}^{\prime }\right)\right]\neq 0 \end{array}$$

Therefore, the elements in the generalized mass matrix *Z* and inertial torque vector *f*^*w*^ can be fully determined.

### 2.4. Determination of Joint Parameters

Identification of joint parameters is different from the previous identification step due to the fact that no motor driven torque can be obtained and that the equivalent joint deformation angle cannot be measured directly. The least-squares method cannot be used to complete the identification because the right term *τ* is zero, which leads to an invalid solution that all parameters are zero. Futhermore, the deformation angles series is not a prescript function of time, so analytical values of angular velocities and accelerations cannot be calculated directly. To address this issue, a motion capture system is introduced to get the motion information of the manipulator, and then the GA method (Chipperfield and Fleming, 1995) is used to identify the joint parameters. Then the problem of parameters identification is converted to an optimization problem of finding the suitable values of *k*_1_, *k*_2_, *c*_1_, and *c*_2_ to minimize an objective function as:

(17) $$O=\frac{\left|{\underline{q}}_{s1}-{\underline{q}}_{1}\right|}{\left|{\underline{q}}_{1}\right|}+\frac{\left|{\underline{q}}_{s2}-{\underline{q}}_{2}\right|}{\left|{\underline{q}}_{2}\right|}$$

where *q*_*si*_, (*i* = 1, 2) is a set of time series of the predicted joint angle of *J*_*i*_, and *q*_*i*_ is the joint angle meausred from simulation or experiment.

**Figure 3:**
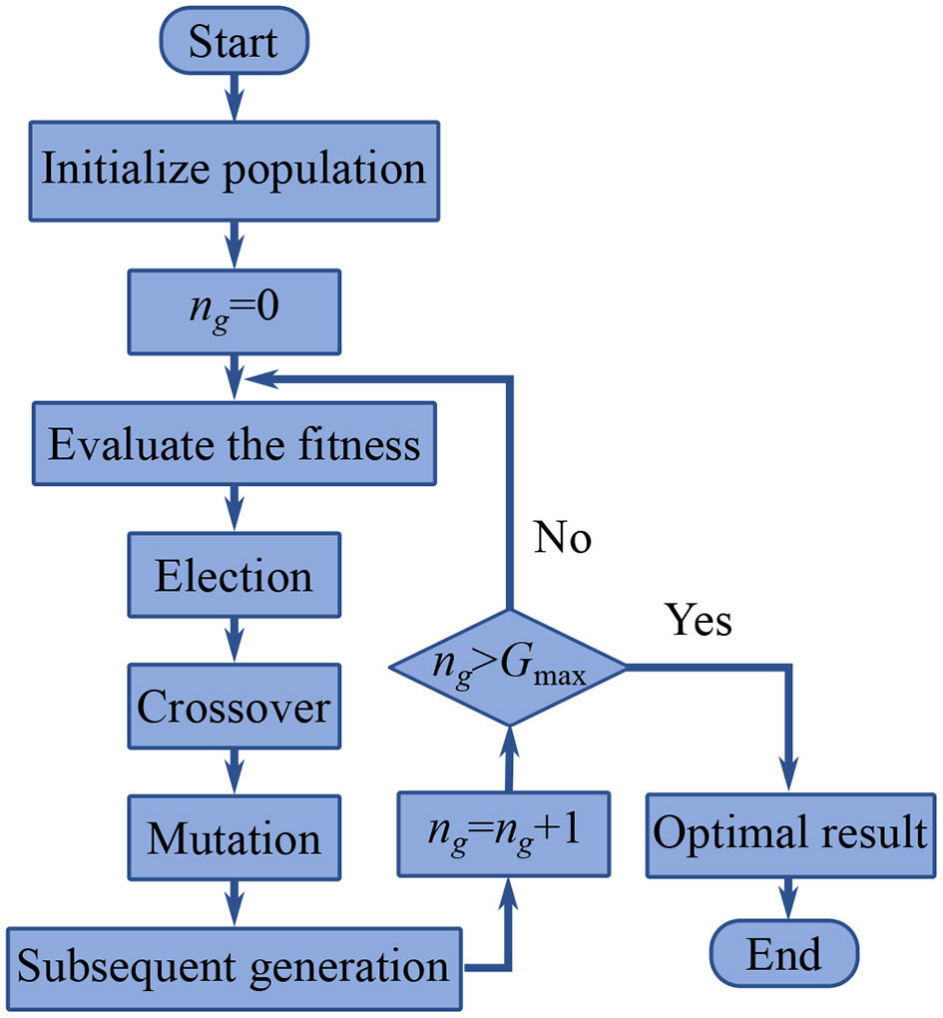
Procedure of the genetic algorithm method.

Figure 3 shows the procedure of the GA method, which iterates around the generational loop until the present generation *n*_*g*_ reaches the default maximum generation *G*_max_ and then terminates. The results of the genetic optimization are the required joint parameters in this step.

### 2.5. Additional Inertia Removing

By using the previous two steps, dynamics parameters of the air bearing supported manipulator system can be fully obtained. The last step to get the true vibration information is to remove the additional inertial caused by the air bearings.

After measuring the mass and inertia of the air bearings, the values of *m*_20_, *I*_20_, *I*_10_, and *m*_20_*ρ*_20_ can be calculated by Equation (5). Consequently, replace the values in Equation (1) with the result above. Dynamics equations of the motor-braked manipulator without air bearings are obtained as:

(18) $${\underline{Z}}_{0}\underline{\ddot{q}}+{\underline{f}}_{0}^{w}+{\underline{f}}^{k}=\underline{0}$$

where

(19) $$\begin{array}{l} {\underline{Z}}_{0}=\left(\begin{array}{cc} I_{10}+I_{20}+m_{20}l_{1}^{2}+2l_{1}m_{20}\left[\rho_{20}\cos\left(\theta_{2,0}+q_{2}\right)\right] & m_{20}\rho_{2}l_{1}\cos\left(\theta_{2,0}+q_{2}\right)+I_{20}\\ m_{20}\rho_{20}l_{1}\cos\left(\theta_{2,0}+q_{2}\right)+I_{20} & I_{20} \end{array}\right) \end{array}$$

and

(20) $$\begin{array}{l} {\underline{f}}_{0}^{w}=\left(\begin{array}{c} m_{20}l_{1}\rho_{2}\left({\dot{q}}_{2}^{2}-{\dot{q}}_{1}^{2}\right)\sin\left(\theta_{2,0}+q_{2}\right)\\ -m_{20}l_{1}\rho_{20}\dot{q_{1}^{2}}\sin\left(\theta_{2,0}+q_{2}\right) \end{array}\right) \end{array}$$

Finally, vibration characteristics can be predicted by Equation (18).

## 3. Validation and Result

In this section, simulations and experiments are designed and conducted to validate the presented procedure of estimating the true vibration characteristics of a tested manipulator. A manipulator capable of operating both with and without air bearings are designed, hence the estimating results can be compared with the measured one directly.

### 3.1. Simulation

**Figure 4:**
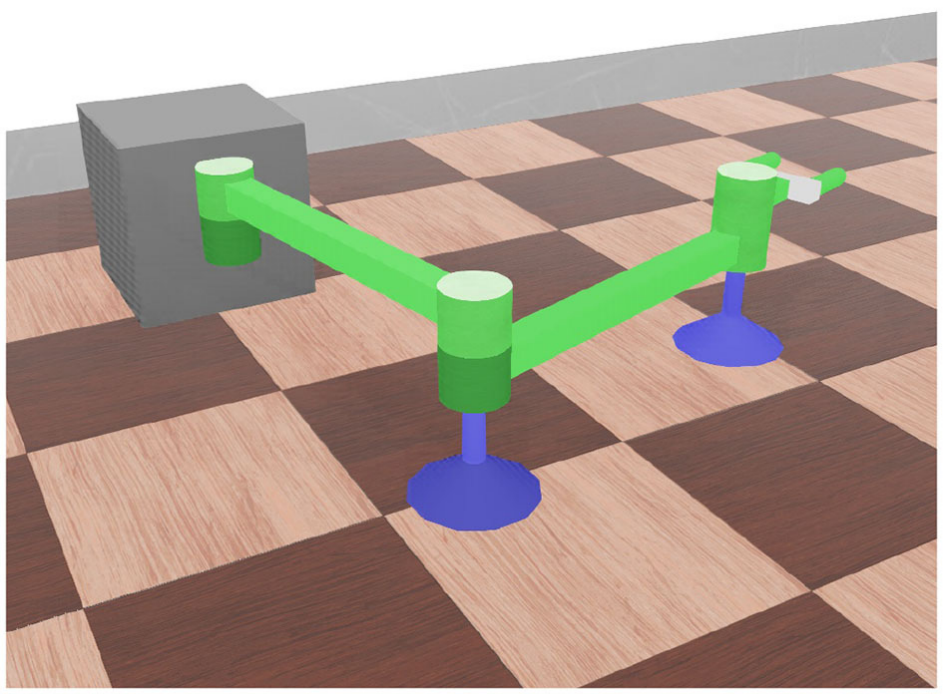
Simulation model of an air bearing supported manipulator.

To illustrate the proposed procedure, a set of numerical simulations is conducted based on the open source platform Webots (Michel, 2004). A no-gravity simulation environment is adopted by assuming that the gravity is counteracted by the air-supporting force. A 3-DOF manipulator is built as shown in Figure 4. Lengths of *L*_1_ and *L*_2_ are both 0.4 m. Joint stiffness is set to be 100 N/rad, and the damping is set to be 0.1 Nms/rad. Other parameters of the simulation model can be found in Table 1.

**Table 1:** Parameters of the simulational manipulator with air bearings.

***l***_**1**_, **m**	***l***_**2**_, **m**	***m***_*s***1**_, **kg**	***m***_*s***2**_	***I***_***s*****1**_ **kg·m**^**2**^	***I***_***s*****2**_ **kg·m**^**2**^
0.4	0.4	1.9635	1.9635	0.00245437	0.00245437

In the first identification step, three tests are conducted according to Equations (10, 11). For Tests I and II, the elbow joint *J*_2_ is locked, and *J*_1_ is driven to perform a trajectory, where *θ*_2, 0_ and $\theta_{2,0}^{\prime }$ are chosen to be 0 and *π*/2, separately. In Test III, *J*_1_ is locked, and *J*_2_ is driven to perform the trajectory. In these tests, the joint trajectory is designed as:

(21) $$\begin{array}{l} \theta =a+b\cos\left(\omega t+\theta_{0}\right) \end{array}$$

where *a* = 0.7854, *b* = –0.7854, *ω* = 0.3, and *theta*_0_ = –0.028. Joint rate and acceleration can be obtained by the first and second derivatives of *θ*:

(22) $$\begin{array}{l} \dot{\theta }=-b\omega \sin\left(\omega t+\theta_{0}\right) \end{array}$$

(23) $$\begin{array}{l} \ddot{\theta }=-b\omega^{2}\cos\left(\omega t+\theta_{0}\right) \end{array}$$

**Figure 5:**
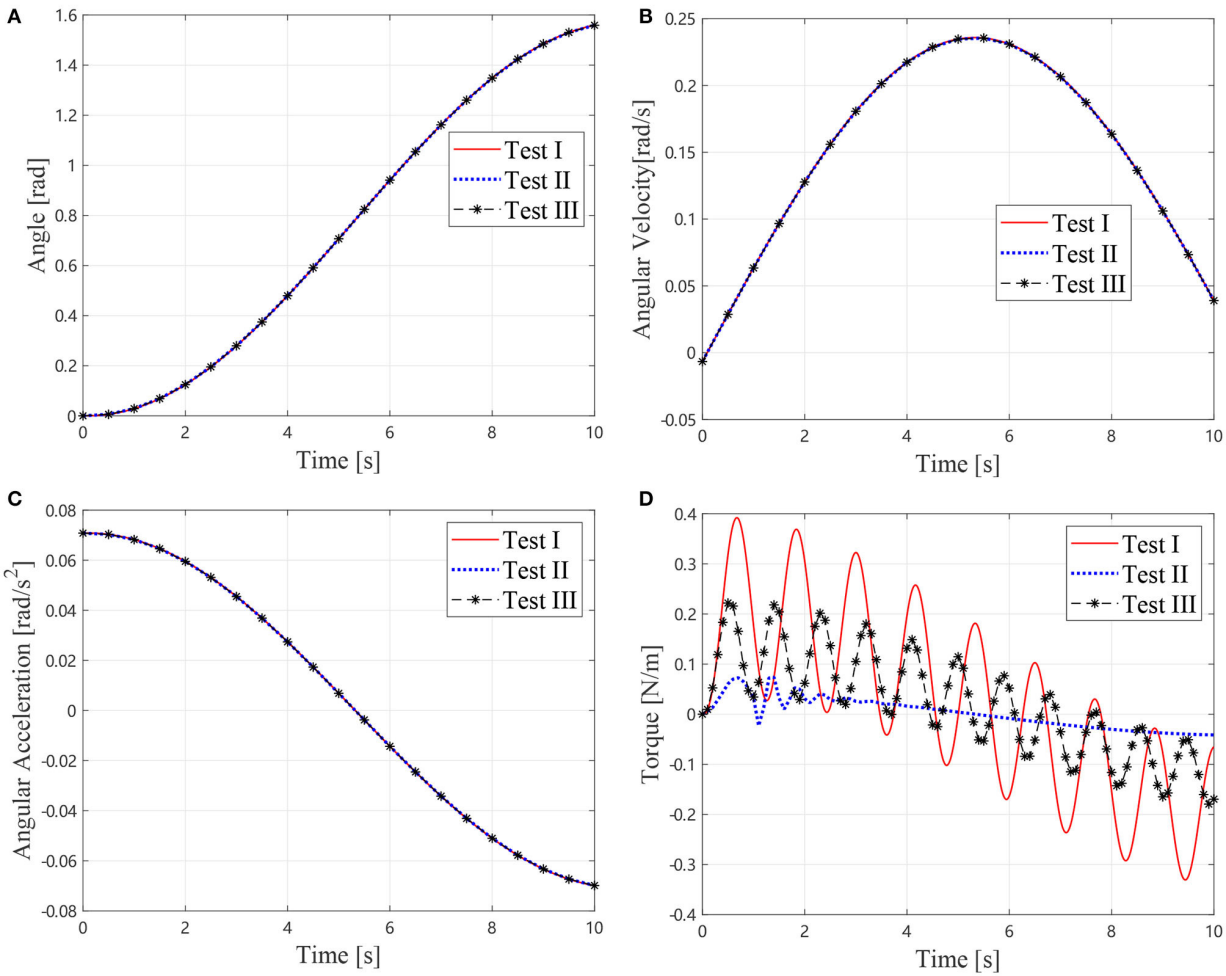
Motions and torque of the simlational Test I, II, and III, **(A)** Joint angles, **(B)** Joint angular velocties, **(C)** Joint angular accelerations, and **(D)** Joint torque.

The joint angle, angular velocity, acceleration, and torque are shown in Figure 5. Parameters of the inertia information are determined as *I*_1_ = 2.8959 kg·m^2^, *I*_2_ = 0.5790 kg·m^2^, and *m*_2_ρ_2_ = 1.4384 kg·m. Consequently, these results can be used in the next step to determine the joint parameters. Comparison between the identification results and the real values are listed in Table 2. The identification error is about 2.3%. It is noticeable that the flexibilities of joint are not considered in the identification procedure, but are considered in the simulation. Therefore, a conclusion can be drawn that the stiffness factor can scarcely influence the identification result, which is because that the stiffness does not dissipate any energy.

**Table 2:** Identification result of the inertial parameters.

	$\left(I_{1}+I_{2}+m_{2}l_{1}^{2}+2m_{2}l_{1}\rho_{2}\right)$	***I***_**2**_
Set value	2.9640	0.5920
Identified value	2.8959	0.5790
Identified error	2.29%	2.20%

The equivalent stiffness of a motor-braked manipulator includes two parts, one is the same as a motor-driven one, and the other is caused by the deformation of the brakes. Therefore, the equivalent stiffness can be treated as a serial of two torsional springs. Then, the stiffness can be calculated by:

(24) $$\begin{array}{l} \frac{1}{k}=\frac{1}{k_{j}}+\frac{1}{k_{b}} \end{array}$$

where *k*_*j*_ is the stiffness caused by joint and link flexibilities and *k*_*b*_ is the stiffness caused by the deformation of the brakes. The value of *k*_*j*_ is set to be the same as the previous simulation, i.e., 100 Nm/rad and *k*_*b*_ are set to be 20 Nm/rad, so the value of the equivalent joint stiffness is 16.67 Nm/rad according to Equation (24).

**Figure 6:**
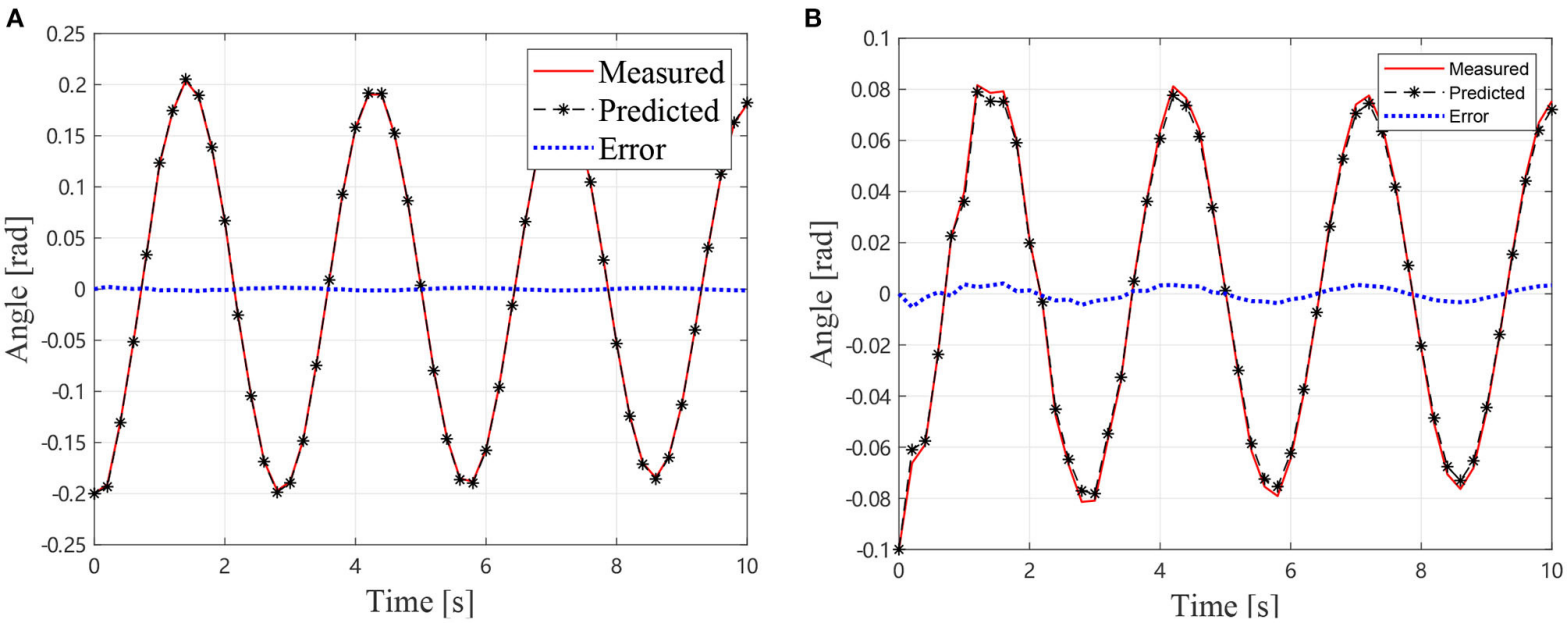
Simulational vibration angles with air bearings, **(A)** Angles of *J*_1_ **(B)** Angles of *J*_2_.

In the GA procedure, 100 individuals are generated in each generation and *G*_max_ is set to be 500. After 500 generations, the values *k*_1_ = 16.2717 Nm/rad, *k*_2_ = 16.1601 Nm/rad, *c*_1_ = 0.0754 Nms/rad, and *c*_2_ = 0.1522 Nms/rad lead to an acceptable agreement with the motion collected from the vibration simulation as shown in Figure 6, which indicates that the first two step is practical to identify the dynamics parameter of an air bearing supported manipulator. In the next section, the identification result is used to predict the motion of the manipulator without air bearings.

**Figure 7:**
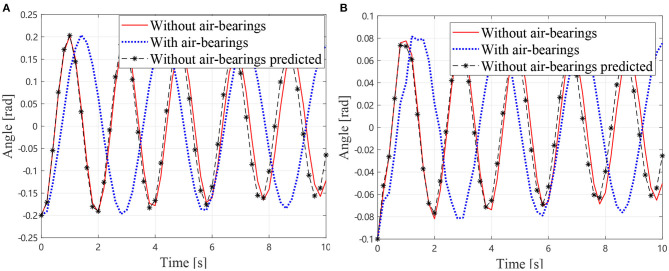
Simulational vibration angles without air bearings, **(A)** Angles of *J*_1_ **(B)** Angles of *J*_2_.

As described in section 2.5, the additional inertia can be removed from the dynamics equation by measuring the mass and inertia of the air bearings. Thus, the dynamics Equation (18) can be solved to predict the motions of the manipulator without air bearings. The predicted joint angles and the measured result without air-bearings (solid lines) and the measured result with air-bearings (dotted lines) are shown in Figure 7. It can be observed that the predicted results are in good agreement with the measured one. The measured frequency of the manipulator without air bearings is 0.5128 Hz. The predicted oscillation frequency of the manipulator without air bearings is 0.5263 Hz, and the measured frequency with air bearings is 0.3488 Hz. Thus, it can be concluded that the predicted value is much closer to the true frequency than the measured one with air bearings. The presented method has reduced the frequency error from 32% of the air bearing test result to 2.6% of the prediction.

### 3.2. Experiment

**Figure 8:**
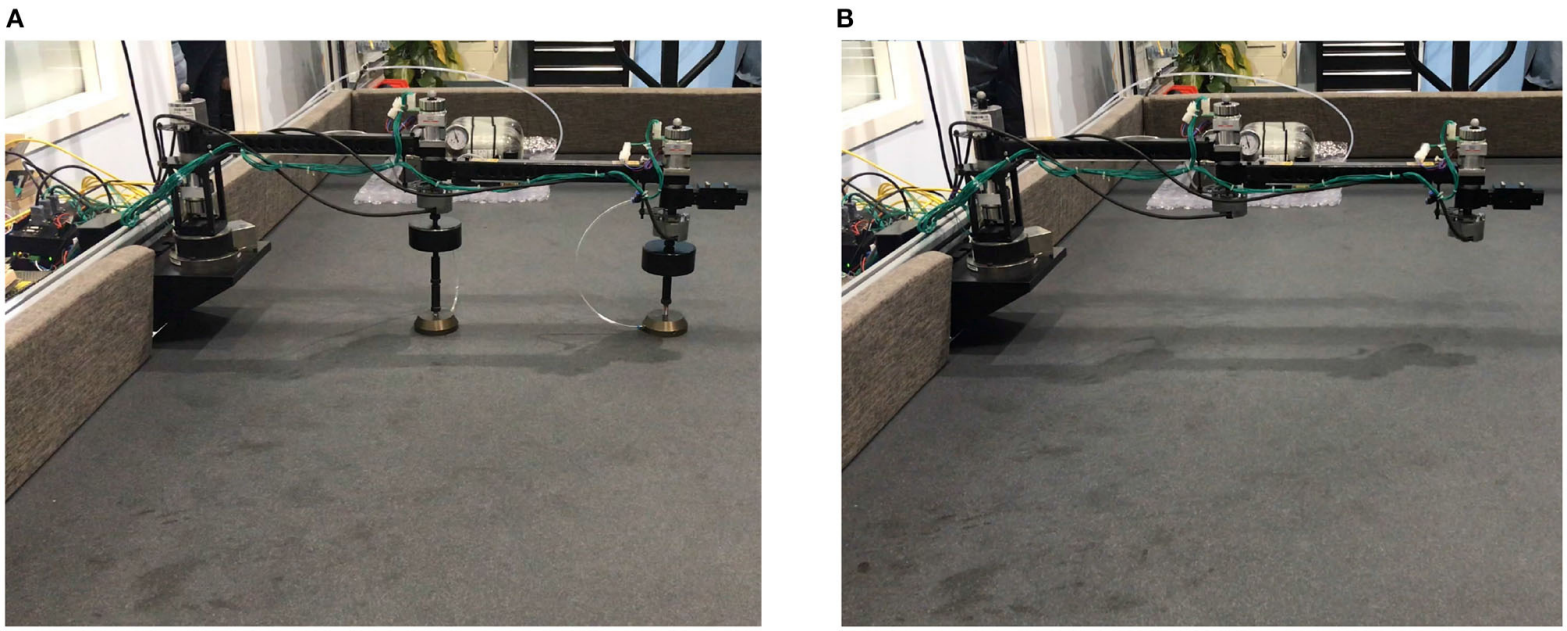
Experiment facilities **(A)** Manipulator with air bearings **(B)** Manipulator without air bearings.

An experimental system is designed and built as shown in Figure 8 to validate the presented method. The test manipulator is the same one used in Yao et al. (2018) which has three single-axis revolute joints, and each joint is actuated by a brushless motor with a harmonic gearbox and an absolute encoder. The manipulator is designed such that it can freely operate on the horizontal plane with (see Figure 8A) or without (see Figure 8B) air bearings. The testing without air bearings represents the true dynamics of the manipulator operating in a microgravity environment. The test data from the testing with air bearings are first treated by the presented procedures and then compared with the data from the testing without air bearings. The comparison result indicates the validity of the presented methods. Markers of a Vicon motion capture system are fixed on the center of each joint. Consequently, the motion of each joint is captured by cameras in the Vicon system. The kinematics parameters of the manipulator and inertial parameters of the air bearings are listed in Table 3.

**Table 3:** Parameters of the experimental manipulator with air bearings.

***l***_**1**_, **m**	***l***_**2**_, **m**	***m***_***s*****1**_, **kg**	***m***_***s*****2**_	***I***_***s*****1**_ **kg·m**^**2**^	***I***_***s*****2**_ **kg·m**^**2**^
0.4	0.4	2.376	2.334	0.001455	0.001430

**Figure 9:**
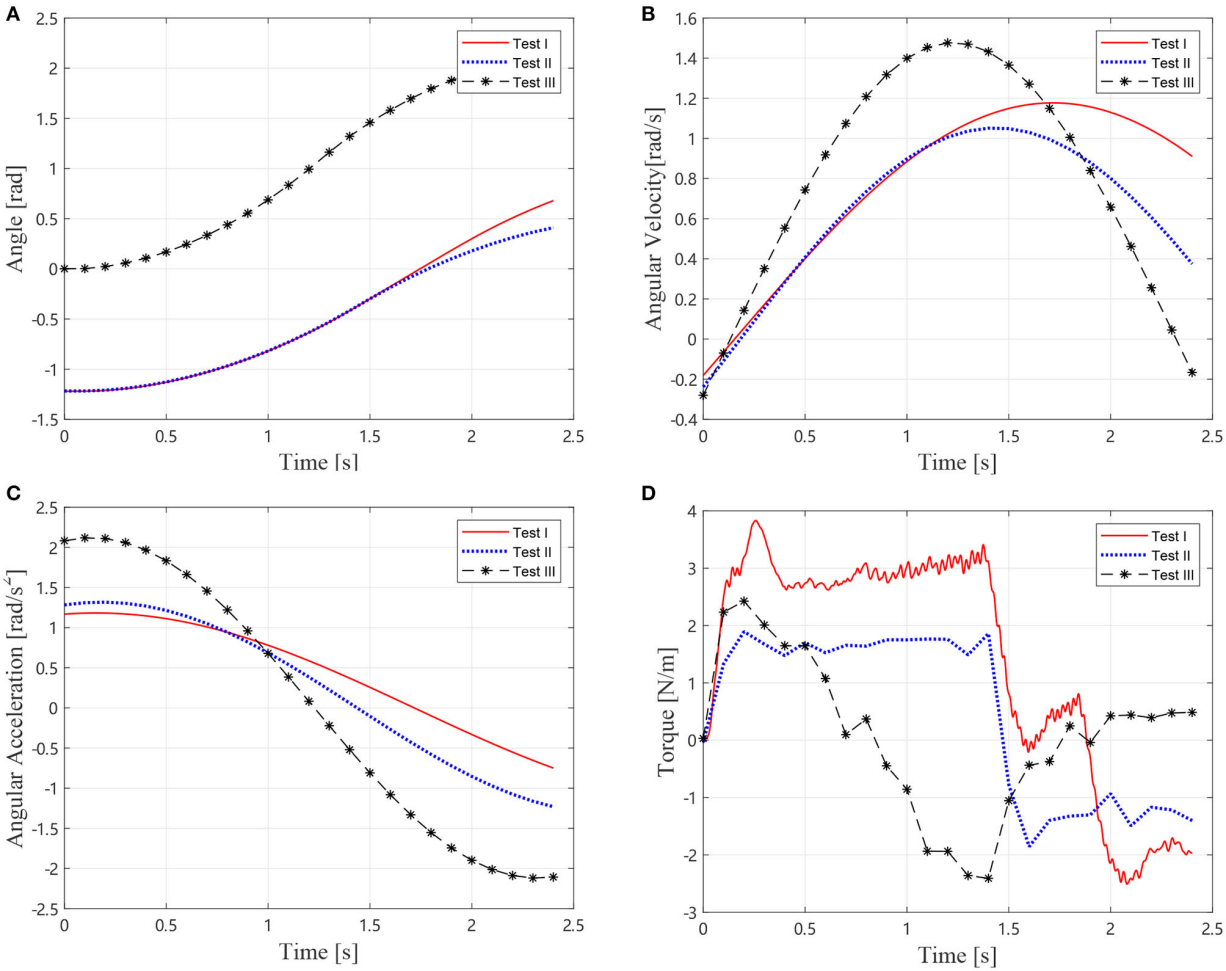
Motions and torque of the experimental Test I, II, and III, **(A)** Joint angles, **(B)** Joint angular velocties, **(C)** Joint angular accelerations, and **(D)** Joint torque.

The same procedure as section 3.1 is conducted on the air bearing platform as shown in Figure 8. In the first identification, three tests were conducted according to Equations (10, 11). For Tests I and II, the elbow joint *J*_2_ is locked, and *J*_1_ is driven according to the trajectory in Figure 9A, where **θ_2, 0_ and $\theta_{2,0}^{\prime }$ are chosen to be 0 and *π*/2, separately. In Test III, *J*_1_ is locked and *J*_2_ is driven to perform the trajectory. The trajectories, angular velocities, accelerations, and torque are shown in Figure 9. Therefore, composition inertia of the manipulator can be identified. The composite inertia $\left[I_{1}+I_{2}+m_{2}l_{1}^{2}+2m_{2}l_{1}\rho_{2}\cos\left(\theta_{2,0}\right)\right]$ in each configuration have been identified by using the results of Tests I and II. The inertia terms *I*_2_ and *m*_2_*ρ*_2_ of *B*_2_ can be determined by solving Equation (10) with the results of Test III.

After the inertial parameters are determined, joint motors are all braked. External forces are applied at the start of the experiment to cause vibrations of the manipulator. Motions of the Vicon markers are collected to calculate the equivalent joint deformation angles.

**Figure 10:**
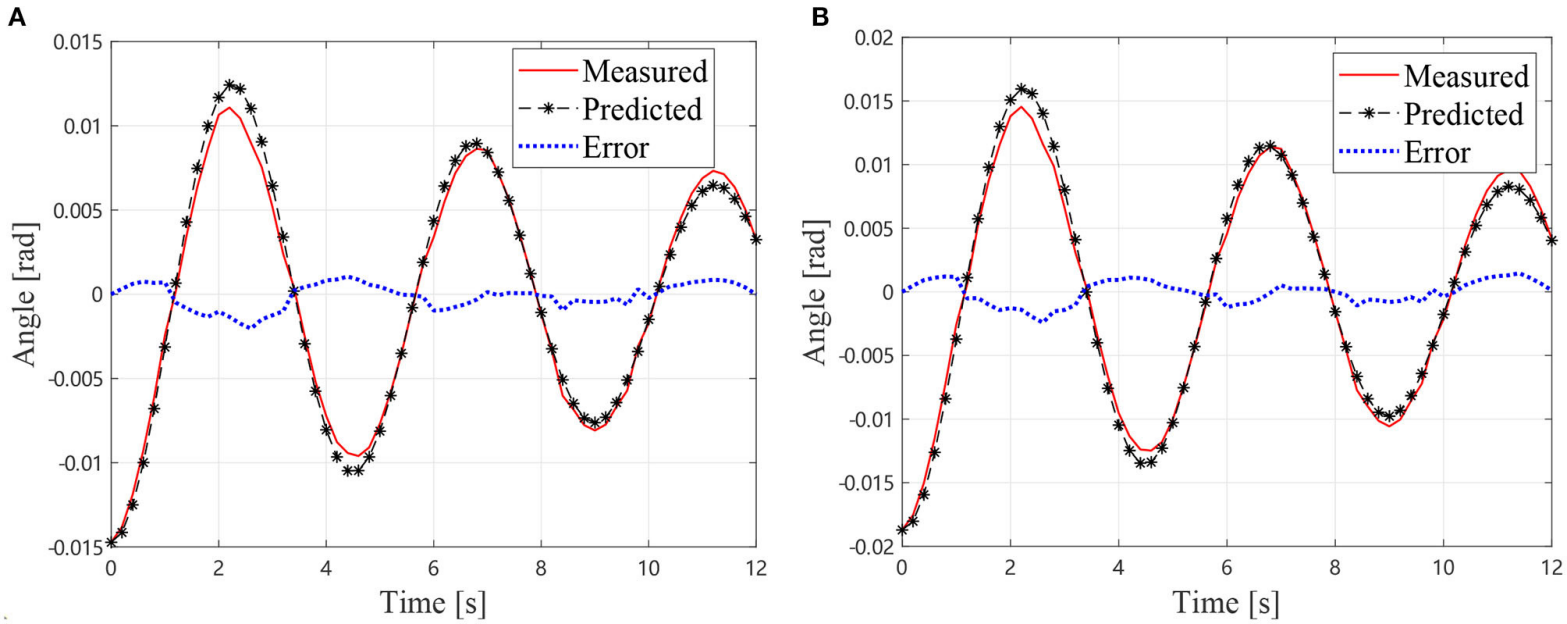
Experimental vibration angles with air bearings, **(A)** Angles of *J*_1_ **(B)** Angles of *J*_2_.

In the GA procedure, 100 individuals are generated in each generation and *G*_max_ is set to be 500. After 500 generations, the values *k*_1_ = 8.30 Nm/rad, *k*_2_ = 3.15 Nm/rad, *c*_1_ = 0.65 Nms/rad, and *c*_2_ = 0.21 Nms/rad lead to an acceptable agreement with the motion collected from the experiment with air bearings as shown in Figure 10, which indicates that the first two steps are practical to identify the dynamics parameter of the air bearing supported manipulator.

In next step, the identification result is used to predict the motion of the manipulator without air bearings.

**Figure 11:**
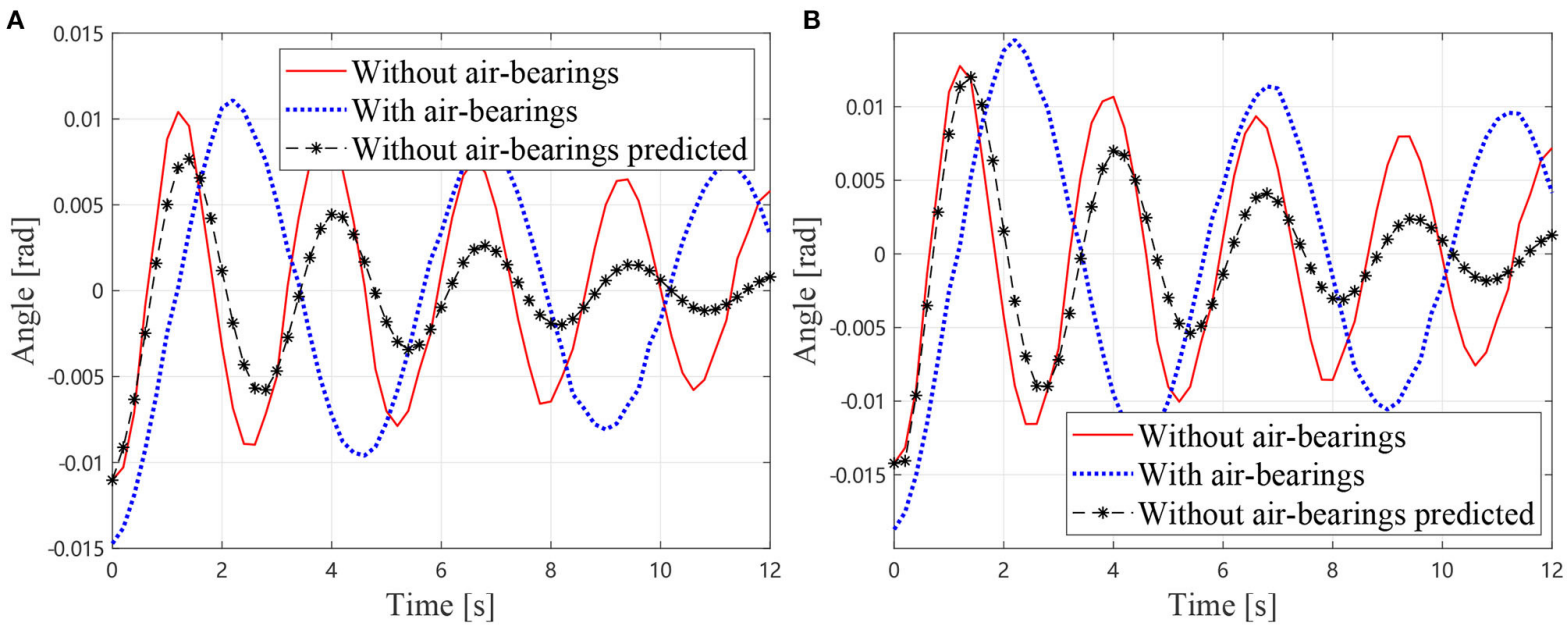
Experimental vibration angles without air bearings **(A)** Angles of *J*_1_ **(B)** Angles of *J*_2_.

As described in section, the additional inertia is removed from the dynamics equation. Thus, the dynamics equation can be solved to predict the motions of the manipulator without air bearings. The predicted joint angles and the measured one of the manipulator without air bearings are shown in Figure 11. It can be observed that the predicted results are in good agreement with the measured one. The measured frequency of the manipulator without air bearings is 0.3750 Hz. The predicted oscillation frequency of the manipulator without air bearings is 0.3390 Hz, and the measured frequency with air bearings is 0.2222 Hz. Thus, it can be concluded that the predicted value is much closer to the true frequency than the measured one with air bearings. The presented method has reduced the frequency error from 40.75% of the air bearing test result to 9.6% of the prediction. Therefore, the presented method for estimating the true vibration frequency of a motor-braked space manipulator from the air bearing supported test data is validated. On the other hand, it is noticeable that the measured result and the predicted one diverging as the time increases due to the fact that the equivalent damping coefficients are much larger than the true values without air bearings. This problem is mainly caused by additional friction and damping of the air bearings. Future work should, therefore, focus on this issue.

## 4. Discussion

This paper presented a three-step procedure to remove additional inertial effect of air bearings from air bearing supported manipulator ground test data. With the presented procedure, true vibration frequency of a joint-braked manipulator can be determined from the air bearing test data. Therefore, the vibration information can be used for on-orbit controller design and the prevention of operational risk. Simulations and experiments are conducted to validate the proposed procedure. Results of the simulations and experiments show that the additional air bearings could introduce a frequency error of about 30 ˜40%. Estimation result of the presented procedure demonstrated that the procedure can process the test data with air bearings close to the true data, and the frequency error is reduced to less than 10%. However, some limitations are worth noting. Although the vibration frequency has been determined by using the presented method, the determined equivalent damping is much larger than the true values without air bearings. This problem is mainly caused by additional friction and damping of the air bearings. Future work should therefore focus on issues such as additional damping of air bearings caused by friction and air resistance.

## Data Availability Statement

The raw data supporting the conclusions of this article will be made available by the authors, without undue reservation.

## Author Contributions

HL developed the program used in this study and wrote most of the manuscript. JL and WR conducted the simulation and experiment. QW led the described research and proposed the main problems. DL and ZT provided the background information for this research. All of the authors proofread the manuscript.

## Conflict of Interest

The authors declare that the research was conducted in the absence of any commercial or financial relationships that could be construed as a potential conflict of interest.
